# Relative effects of mutability and selection on single nucleotide polymorphisms in transcribed regions of the human genome

**DOI:** 10.1186/1471-2164-9-292

**Published:** 2008-06-17

**Authors:** Ivan P Gorlov, Olga Y Gorlova, Christopher I Amos

**Affiliations:** 1 Department of Epidemiology, The University of Texas M. D. Anderson Cancer Center, Houston, Texas 77030, USA

## Abstract

**Motivation:**

Single nucleotide polymorphisms (SNPs) are the most common type of genetic variation in humans. However, the factors that affect SNP density are poorly understood. The goal of this study was to estimate the relative effects of mutability and selection on SNP density in transcribed regions of human genes. It is important for prediction of the regions that harbor functional polymorphisms.

**Results:**

We used frequency-validated SNPs resulting from single-nucleotide substitutions. SNPs were subdivided into five functional categories: (i) 5' untranslated region (UTR) SNPs, (ii) 3' UTR SNPs, (iii) synonymous SNPs, (iv) SNPs producing conservative missense mutations, and (v) SNPs producing radical missense mutations. Each of these categories was further subdivided into nine mutational categories on the basis of the single-nucleotide substitution type. Thus, 45 functional/mutational categories were analyzed. The relative mutation rate in each mutational category was estimated on the basis of published data. The proportion of segregating sites (PSSs) for each functional/mutational category was estimated by dividing the observed number of SNPs by the number of potential sites in the genome for a given functional/mutational category. By analyzing each functional group separately, we found significant positive correlations between PSSs and relative mutation rates (Spearman's correlation coefficient, at least r = 0.96, df = 9, *P *< 0.001). We adjusted the PSSs for the mutation rate and found that the functional category had a significant effect on SNP density (F = 5.9, df = 4, *P *= 0.001), suggesting that selection affects SNP density in transcribed regions of the genome. We used analyses of variance and covariance to estimate the relative effects of selection (functional category) and mutability (relative mutation rate) on the PSSs and found that approximately 87% of variation in PSS was due to variation in the mutation rate and approximately 13% was due to selection, suggesting that the probability that a site located in a transcribed region of a gene is polymorphic mostly depends on the mutability of the site.

## Background

Single nucleotide polymorphisms (SNPs) are the most common type of genetic variation in humans, accounting for approximately 90% of genetic variations and occurring every 400 nucleotides on average [1,2]. SNPs are believed to be the major contributors to interindividual genetic variation in susceptibility to common human diseases [3-6]. They are distributed nonrandomly: in some regions, their density is high, and in others, it is low [2,7-9]. Because the exonic regions are most likely to carry functional polymorphisms [10], it is important to identify factors that affect SNP density in exons.

The results of several studies suggest that purifying selection affects SNP density [11,12]. A comparison of the fixation rates for synonymous, nonsynonymous, and disease-associated mutations reveals that negative selection operates against nonsynonymous SNPs [13-15]. Different SNP types are expected to vary in the intensity of negative selection against them; for example, missense mutations are expected to be more deleterious than are silent mutations [16,17].

A few studies have demonstrated that the mutation rate also can affect SNP density. Schmegner *et al*. [18] compared SNP densities in G+C-poor and G+C-rich regions and found a higher SNP density in G+C-rich (i.e., more mutable) regions, suggesting that SNP density is correlated with mutation rate. Horvath *et al*. [19] found that G > A transitions that occur at CpG sites and produce silent mutations were the most common substitution, suggesting that both mutability and selection play roles in SNP density in the coding regions of the human genome. However, the relative effects of these two factors have never been evaluated on a genome-wide scale. The goal of this analysis was to estimate the relative effects of mutability and selection on SNP density. Here we estimate the proportion of segregating (polymorphic) sites in different regions. The effect of selection is estimated by evaluating the differences in the proportion of segregating sites between synonymous, nonsynonymous SNP, as well as 5' UTR and 3' UTR potential sites. The effect of mutation rate is estimated by looking at difference in the proportions of segregating sites for single nucleotide substitutions that differ by mutation rate (e.g. CpG vs. non-CpG sites).

## Methods

### SNP data retrieval

We searched the dbSNP database BUILD128 [20-22] to identify SNPs. The database was accessed February, 2008. To reduce discovery errors, only validated SNPs (i.e., those submitted by at least two independent researchers, with at least one submission validated by a noncomputational method) were used in this study.

SNPs were stratified into five functional categories: (i) 5' untranslated region (UTR) SNPs, (ii) 3' UTR SNPs, (iii) synonymous SNPs, (iv) SNPs producing conservative missense mutations, and (v) SNPs producing radical missense substitutions. Missense substitutions were further stratified as radical or conservative according to criteria suggested by Zhang [23]. Briefly, all amino acids were subdivided into three groups according to their charge: positive (R, H, K), negative (D, E) and uncharged (A, N, C, Q, G, I, L, M, F, P, S, T, W, Y, V). The amino acids were further subdivided by volume and polarity: special (C), neutral and small (A, G, P, S, T), polar and relatively small (N, D, Q, E), polar and relatively large (R, H, K), non-polar and relatively small (I, L, M, V), and non-polar and relatively large (F, W, Y). We considered radical missense mutations to be those that change amino acid categories (e.g. R→L) and conservative missense mutations to be those that do not change amino acid category (e.g. L→V).

### Accounting for overlapping genes

Approximately 3% of genes in the human genome overlap [24], suggesting that some SNPs can be categorized differently depending on the overlapping gene analyzed. In this study, we identified 96, or 0.4% of the total number of SNPs used in the analysis that were located in overlapping genes. Overlapping SNPs were counted separately for each overlapping gene. For example, rs3736360 is a radical missense mutation in the HSPG2 gene and it is a 3' UTR SNP in the context of the overlapping gene LDLRAD2. We have conducted analyses both including and excluding the overlapping SNPs. The results of the analyses were essentially the same, because of the very low proportion of excluded SNPs. Because multiple counting of the same SNPs violates the assumption of independent data points, we excluded SNPs located in overlapping genes from our final analyses.

### Ancestral and derived alleles

We used data on ancestral and derived alleles from the dbSNP database [22,25] and the Haplotter database [26]. The requirements that SNPs should be frequency-validated and that their ancestral state should be known limited the number of SNPs that could be used in the analysis. The number of analyzed SNPs, subdivided by categories, were 1424 for 5' UTR SNPs, 7705 for 3' UTR SNPs, 6972 for synonymous SNPs, 2567 for conservative missense mutations, and 3528 for radical missense mutations.

Knowing the ancestral and derived alleles does not tell us on which DNA strand the substitution has occurred. For example, for a C/T SNP with ancestral allele C, the ancestral pair of nucleotides is C:G and the derived pair of nucleotides is T:A. Two substitutions are possible in this case, C > T or G > A. Because it is impossible to tell these two substitutions apart, we analyzed them jointly. We separately analyzed substitutions located in CpG sites. Therefore, the total number of mutational categories analyzed in this study was nine: (1) nonCpG (C:G > A:T), (2) nonCpG (C:G > G:C), (3) nonCpG (C:G > T:A), (4) CpG (C:G > A:T), (5) CpG (C:G > G:C), (6) CpG (C:G > T:A), (7) (A:T > C:G), (8) (A:T > G:C), and (9) (A:T > T:A). Each was stratified into five subgroups according to the position in the coding region and type of mutation (functional types), resulting in 45 functional/mutational categories.

### Estimating relative mutation rates

To estimate the relative mutation rates, we used mutation rates obtained by an analysis of processed pseudogenes [27,28] and direct estimates of mutation rates derived from an analysis of mutations causing Mendelian diseases in humans [29]. The results of these studies agree closely: the Pearson's correlation coefficient between the two estimates is 0.997 (Additional file 1). We assumed that the mean mutation rate for transversions in nonCpG sites (as the lowest mutation rate) equals one and computed the relative mutation rates for other types of SNPs. To estimate the transition rates in CpG sites, we used the ratio of CpG to nonCpG transitions determined by [28]. We used a similar method to estimate the relative mutation rates for CpG and nonCpG C > A, C > G, G > C, and G > T transversions (Table 1). The original data and computations are presented in Additional file 1. Relative mutation rates were estimated by assuming that mutation rates for C > A and G > T transversions are equal one each; then, given two transversions, it is two for the pair of the transversions. Relative mutation rates for all other substitutions were computed based on the published estimates of the mutation rates (see Additional table 2 for details). All these numbers can be scaled by dividing by two.

**Table 1:** Estimated relative mutation rates for pairs of reciprocal substitutions^a^

Transition/Transversion	CpG	Substitution pair	RMR
Transversion	nonCpG^b^	C:G > A:T	2.0
Transversion	nonCpG^b^	C:G > G:C	2.0
Transition	nonCpG	C:G > T:A	7.3
Transversion	CpG	C:G > A:T	16.0
Transversion	CpG	C:G > G:C	16.0
Transition	CpG	C:G > T:A	96.7
Transversion	nonCpG	A:T > C:G	2.2
Transition	nonCpG	A:T > G:C	7.3
Transversion	nonCpG	A:T > T:A	1.8

^a^Original data and computing of relative mutation rates can be found in Additional file 1.

^b^Relative mutation rate is one per transversion C > A or G > T giving 2 for the C:G > A:T pair

### Estimating number of potential sites for each functional/mutational category

A potential site was defined as a single nucleotide substitution (SNS) that would produce a SNP of a specific functional/mutational category. In each nucleotide position, three SNSs are possible, which correspond with three potential sites; therefore, the total number of potential sites per codon is nine.

To estimate the number of potential sites in the coding, 3' UTR, and 5' UTR regions, we used data from the codon usage database [30]. We considered nine possible SNSs in each of 64 codons, yelding a total of 576 wild type-mutant codon pairs. The pairs were classified as radical nonsynonymous, conservative nonsynonymous, synonymous, or other (nonsense mutations and mutations that change a stop codon into an amino acid-encoding codon [elongating]). These types of codon pairs were not used in this analysis because corresponding SNPs are relatively rare and have a high discovery-error rate [31]. To estimate the numbers of potential sites in these regions, we used data on the nucleotide content of 5' UTR and 3' UTR regions [32,33].

The National Center for Biotechnology Information Entrez Gene database (accessed February, 2008) contains about 23,000 known genes. On the basis of the estimated mean size of the 5' UTR region, 300 bp, the total number of potential sites in 5' UTR region is approximately 20,700,000 (23,000 × 300 × 3). Similarly, on the basis of the estimated size of the 3' UTR region, 770 bp, the total number of potential sites in the human genome for the 3' UTR region is 53,130,000.

On the basis of the mean number of exons in a gene (8.8), the mean size of the exon (145 nucleotides) [34,35], and the number of known genes ~23,000 (based on the NCBI Entrez Gene accessed April 30, 2008), the total number of potential sites in the coding region is 88,044,000.

### Estimating number of potential sites in CpGs

Because the mutation rate in CpG dinucleotides is higher than that in nonCpGs [36-40], they were analyzed separately. To estimate the number of potential sites located in CpGs, we first estimated the proportions of synonymous, radical, and conservative missense mutations resulting from SNSs in CpG-containing codons. To account for CpGs on codon boundaries, we flagged codons that started with G or ended with C. The proportion of Gs in the first codon position was 0.31, and the proportion of Cs among the third positions was 0.30. Assuming a random combination of codons, the probability that the first G nucleotide in a codon would be in a CpG site was 0.30, and the probability that the last C in a codon would be in a CpG site was 0.31. The product of these frequencies and the frequency of the corresponding codon gives the expected proportion of boundary-located CpGs. To account for possible violation of the assumption on the random combination of nucleotides on codon boundaries we compared the expected and the observed number of CpG on codon boundaries in 56 Seattle genes (see section 2.7 for the list of the genes). The expected proportion of the CpGs on codon boundaries was 0.072 and the observed 0.023 suggesting that there is the observed number of CpGs on codon boundaries is ~3.1 times lower compared to the expected one. We correspondingly reduced the expected number of the boundary-located CpGs by the factor 3.1.

### SeattleSNPs dataset

The SeattleSNPs project generated SNP data for samples from both European and African populations [41]. The database contains sequencing data from genes that are likely to play a role in common human diseases. The sample size includes 24 African descent and 23 European descent subjects. The SNPs were identified by genomic DNA sequencing and, therefore, provide unbiased representation of different types of SNPs in gene regions. We analyzed the SNPs detected in 56 completely sequenced genes: *AGTRAP, ALOX15, BF, C1QA, CCR2, CSF2, CSF3, CSF3R, CXCR4, F12, F2RL1, F2RL2, F9, FGA, FGG, FGL2, FSBP, GP1BA, ICAM4, IFNG, IL10, IL10RA, IL13, IL16, IL17, IL17B, IL19, IL1B, IL1R1, IL1R2, IL1RN, IL2, IL20, IL22, IL3, IL4, IL5, IL7R, IL9, IRAK4, KEL, LTA, LTB, NFKBIA, PCSK9, PFC, PTGS2, SFTPA2, SFTPC, SFTPD, TCF1, TNF, VCAM1, VEGF, VTN, ZNF202*. We excluded deletions, insertions, and sites with more than 2 alleles from the analysis. We subdivided all SNPs into 6 functional categories: (i) 5' untranslated region (UTR) SNPs, (ii) 3' UTR SNPs, (iii) synonymous SNPs, (iv) SNPs producing conservative missense mutations, (v) SNPs producing radical missense substitutions, and (vi) SNPs in intronic regions. We included SNPs from the intronic regions because, in the contrast to dbSNP database, sequencing of the genomic DNA provides an unbiased estimated of SNPs in the genic region. The number of potential sites for the functional/mutational categories was estimated based on sequence of the 56 SeattleSNPs genes. The sequences 5' UTR, 3' UTR, intronic and CDS regions were retrieved using the University of California Santa Cruz (UCSC) Genome Browser (accessed February 12, 2008). Table 2 shows the sizes and nucleotide compositions of 5' UTR, 3' UTR, intronic and CDS regions.

**Table 2:** The total size and nucleotide compositions of the regions from the 56 completely sequenced genes from the SeattleSNP database

Region	Size (nc)	A	C	G	T	CpG
5'UTR	7768	1770	2081	1938	1575	404
CDS*	64295	15454	14904	14492	17819	1626
Intronic	557915	150596	116032	121989	163818	5480
3'UTR	47900	13601	9850	9703	14266	480

* coding sequence

### Statistical analysis

The proportion of segregating sites (PSSs) was estimated as the ratio of frequency-validated SNPs of a specific functional/mutational category to the number of potential sites for that category in the human genome. An analysis of variance (ANOVA) was used to estimate the effect of functional categories. An analysis of covariance (ANCOVA) was used to estimate the effect of the mutation rate on the PSS, controlling for functional category. All statistical analyses were performed using Statistica (StatSoft, Inc., Tulsa, OK, USA).

## Results

Table 3 shows the estimated absolute numbers of potential sites. The total number of potential sites for SNPs in coding regions was estimated to be 9.8 × 10^7^. That is less than the expected number (1.02 × 10^8^), which was based on the size of the coding region of the human genome, 34 × 10^6 ^nucleotides [35]. This inconsistency occurs because SNPs that produce nonsense and protein-elongating mutations were excluded from the analysis. There was nearly 40-fold variation in the number of potential sites for the various functional/mutational categories. The smallest number of potential sites was observed in CpG dinucleotides in the 3' UTR region; this number was small simply because the frequency of CpG sites is low in this region. The maximal number of potential sites was for nonCpG A:T > C:G substitutions producing radical missense mutations.

**Table 3:** Number of potential sites for the 45 functional/mutational categories of SNPs in the human genome

CpG	Substitution	5' UTR	3' UTR	Rad	Cons	Syn
nonCpG	C:G > A:T	3924000	8778000	8216939	2632167	2456534
nonCpG	C:G > G:C	3924000	8778000	7856924	3992722	2291532
nonCpG	C:G > T:A	3924000	8778000	5578380	2520193	5495499
CpG	C:G > A:T	187200	110880	621000	168400	439500
CpG	C:G > G:C	187200	110880	882400	147300	329100
CpG	C:G > T:A	187200	110880	520400	293900	538100
nonCpG	A:T > C:G	2808000	9424800	10917400	2317667	2610067
nonCpG	A:T > G:C	2808000	9424800	8022867	3278733	4780400
nonCpG	A:T > T:A	2808000	9424800	9833933	2457067	2523933

		20757600	54941040	52450243	17808149	21464665

						167421697

Table 4 shows the observed number of SNPs for the functional/mutational categories. The lowest number of SNPs was found for C:G > A:T substitution in CpGs producing conservative missense mutations, and the highest number was found for A:T > G:C substitutions in the 3' UTR region. The difference between the lowest and highest numbers of observed SNPs was 200-fold.

**Table 4:** Observed number of SNPs in each functional/mutation category (dbSNP data)

CpG	Substitution	5' UTR	3' UTR	Rad	Cons	Syn
nonCpG	C:G > A:T	108	531	268	190	187
nonCpG	C:G > G:C	139	639	293	365	275
nonCpG	C:G > T:A	353	1830	736	502	1954
CpG	C:G > A:T	74	65	49	25	155
CpG	C:G > G:C	30	56	61	25	67
CpG	C:G > T:A	275	1341	926	554	2171
nonCpG	A:T > C:G	76	522	261	105	230
nonCpG	A:T > G:C	321	2336	796	698	1790
nonCpG	A:T > T:A	48	383	138	103	142

Total		99	649	408	222	464

Table 5 shows data on the proportions of segregating sites (PSSs) in 45 functional/mutational categories. The mean PSS was approximately 2.8 × 10^-6^. The highest PSS detected among the potential sites (4.8 × 10^-3^) was for CpG C:G > T:A substitutions in the 3' UTR region; the lowest (1.4 × 10^-5^) was for nonCpG A:T > T:A substitutions producing radical missense mutations. To evaluate how PSS depends on the relative mutation rate, we estimated the correlation between PSSs and relative mutation rates. An analysis conducted within functional categories yielded Pearson's correlation coefficients that varied from 0.95 (for radical missense mutations) to 0.99 (for SNPs located in the 3' UTR region). All correlation coefficients were significant (P < 0.0001). To remove the effect of the mutation rate, we divided PSSs by the corresponding mutation rate (Table 6). After this adjustment, the variation in PSSs was considerably lower. We used ANOVA to estimate the effect of functional categories on adjusted PSSs and found that the effect of the functional category was significant (F = 5.9, df = 4, *P *= 0.001).

**Table 5:** Proportion of segregating sites (PSS) in the 45 functional/mutational categories^a^

CpG	Substitution	5' UTR	3' UTR	Rad	Cons	Syns
nonCpG	C:G > A:T	2.7	6.1	3.3	7.2	7.6
nonCpG	C:G > G:C	3.6	7.3	3.7	9.1	12.0
nonCpG	C:G > T:A	9.0	20.8	13.2	19.9	35.6
CpG	C:G > A:T	15.8	23.6	3.2	5.9	14.1
CpG	C:G > G:C	6.4	20.4	2.8	6.8	8.1
CpG	C:G > T:A	58.8	483.8	71.2	75.4	161.4
nonCpG	A:T > C:G	2.7	5.5	2.4	4.5	8.8
nonCpG	A:T > G:C	11.4	24.8	9.9	21.3	37.5
nonCpG	A:T > T:A	1.7	4.1	1.4	4.2	5.6

^a^All values × 10^5^

**Table 6:** Proportion of segregating sites adjusted for relative mutation rates^a^

CpG	Substitution	5' UTR	3' UTR	Rad	Cons	Syn
nonCpG	C:G > A:T	1.4	3.0	1.6	3.6	3.8
nonCpG	C:G > G:C	1.8	3.6	1.9	4.6	6.0
nonCpG	C:G > T:A	1.2	2.9	1.8	2.8	4.9
CpG	C:G > A:T	1.0	1.5	0.2	0.4	0.9
CpG	C:G > G:C	0.4	1.3	0.2	0.4	0.5
CpG	C:G > T:A	0.6	5.0	0.7	0.8	1.7
nonCpG	A:T > C:G	1.3	2.6	1.1	2.2	4.2
nonCpG	A:T > G:C	1.6	3.4	1.4	3.0	5.2
nonCpG	A:T > T:A	0.9	2.3	0.8	2.3	3.1

^a^All values × 10^6^

**Figure 1:**
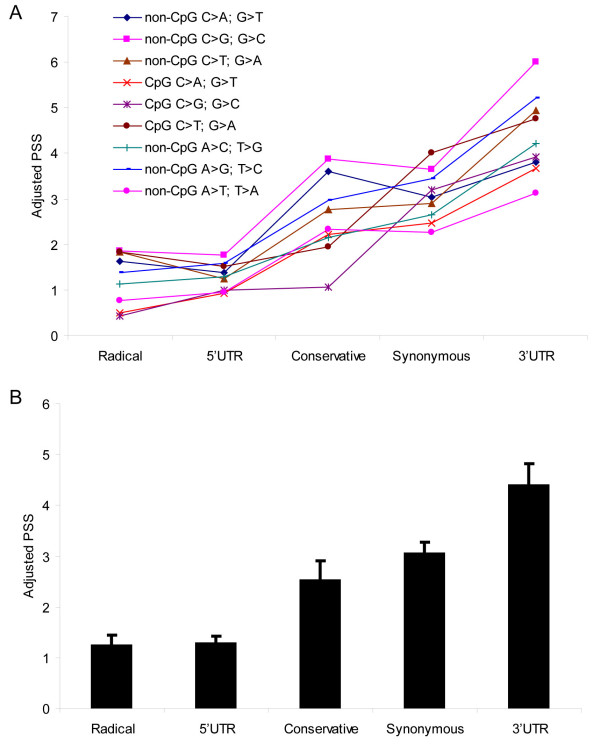
**PSSs adjusted for mutation rates (dbSNP data).A) **PSSs (× 10^6^) for the 45 functional/mutational categories. **B) **Mean PSSs among five functional categories.

Figure 1A shows the PSSs for nine mutational categories after the adjustment for the relative mutation rate. We did not observe significant differences in the adjusted PSSs between different mutational categories. The lowest average adjusted PSS was found for radical missense mutations (1.1 × 10^-5^), and the highest – for 3' UTR region (3.4 × 10^-5^) (Figure 1B).

To estimate the relative effects of mutation rate and selection on PSS, we compared a linear model that used both the mutation rate and functional type as predictors (model 1) with two others that used only the mutation rate (model 2) or functional type (model 3) as the predicting variable. Because PSS data were not normally distributed, we used log transformation to normalize data before conducting statistical tests (an analysis of nontransformed data produced similar results, data not shown). We used ANCOVA for model 1, with PSSs as the outcome variable, mutation rate as the continuous predictor, and functional type as the categorical predictor. The whole-model *P *value was <0.001, with R^2 ^= 0.53 (F = 8.9, df = 5, P < 0.001). Model 2 was based on univariate linear regression, with only mutation rate as the predictor. In this model, R^2 ^was equal to 0.46 (F = 35.6, df = 1, P < 0.001). Model 3 was based on the ANOVA, with functional type as the categorical predictor. The R^2 ^for this model was equal to 0.07 (F = 3.3, df = 4, *P*<0.03). Summation of R^2^s for second and third model gives the expected 53% of variation explained by model 1 that includes both functional categories and the relative mutation rate as predictors. These estimates also demonstrate that 87% [(0.53–0.07)/0.53 = 0.87] of the variation in the proportion of polymorphic sites was due to variations in mutation rate and 13% was due to the effect of functional category that was used to assess the effect of purifying selection.

**Figure 2:**
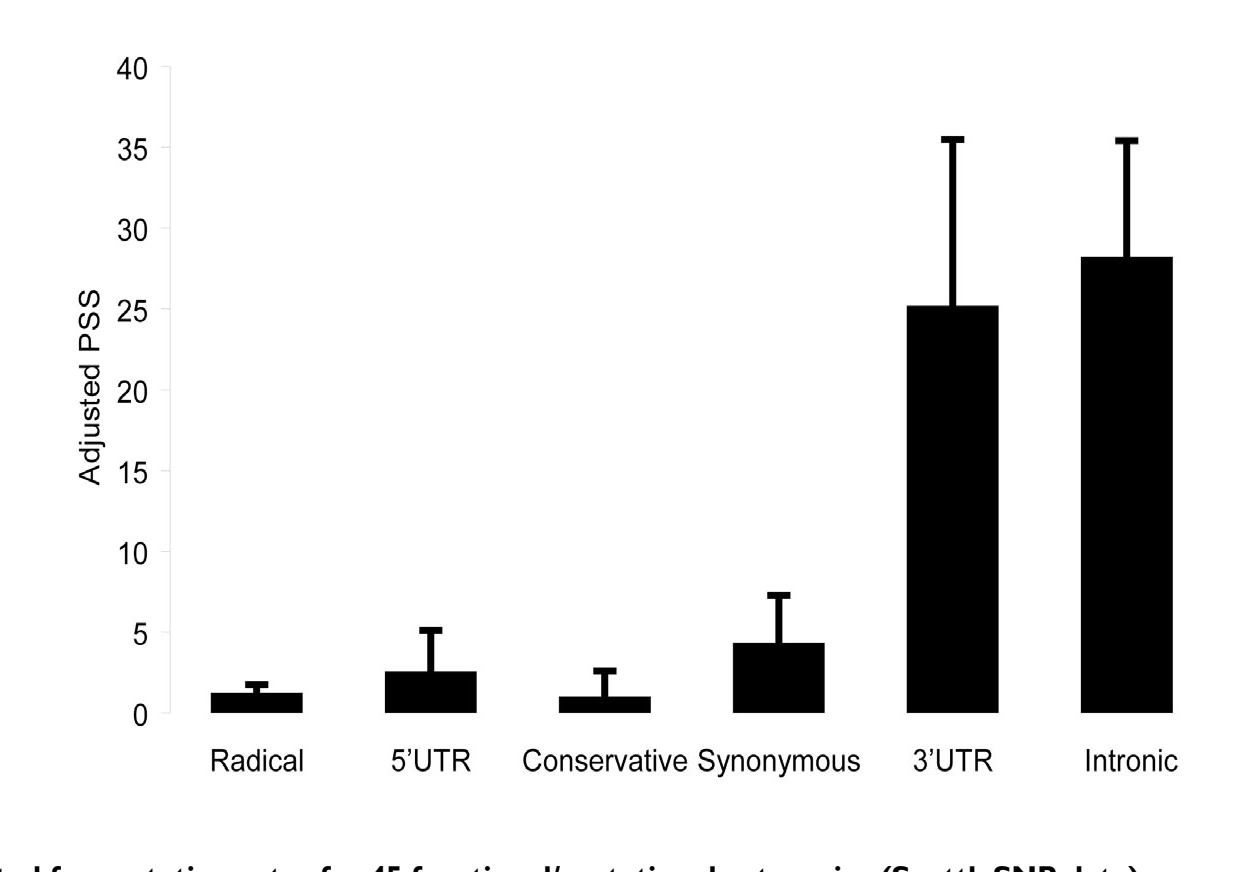
PSSs adjusted for mutation rates for 45 functional/mutational categories (SeattleSNP data).

Analysis of SeattleSNPs produced results that are similar to what we have found by the analysis of the dbSNP data. The total number of SNPs from the Seattle genes was 2474 with 11 SNPs detected in 5' UTR region, 139 SNPs detected in 3' UTR region, 176 SNPs producing radical, and 111 SNPs producing conservative missense mutations, 306 synonymous, and 1731 SNPs detected in intronic regions. The number of SNPs in each of the 45 functional/mutational categories and the estimated number of potential sites for each category is shown in Table 7. Figure 2 shows the adjusted proportions of the segregating sites in the six functional categories of SNPs. Table 8 shows the adjusted PSSs for the SeatlleSNPs data. The adjusted PSSs for SNPs in the coding was significantly lower compared to the 3' UTR and intronic SNPs with P-values less than 0.01. No difference in the adjusted PSSs between SNPs in the coding regions and SNPs from the 5' UTR region were found probably because of the small sample size: the number of the 5' UTR SNPs was 11.

**Table 7:** Observed number of SNPs in each functional/mutation category (SeattleSNP data)

CpG	Substitution	5' UTR	3' UTR	Rad	Cons	Syn	Introns
nonCpG	C:G > A:T	0	10	15	14	12	113
nonCpG	C:G > G:C	0	7	17	16	9	143
nonCpG	C:G > T:A	4	33	38	24	69	385
CpG	C:G > A:T	0	0	2	1	12	6
CpG	C:G > G:C	0	2	7	0	6	20
CpG	C:G > T:A	3	23	44	23	123	292
nonCpG	A:T > C:G	2	12	11	6	6	125
nonCpG	A:T > G:C	2	42	35	26	69	539
nonCpG	A:T > T:A	0	10	7	1	0	108

Total		11	139	176	111	306	1731

**Table 8:** Proportion of segregating sites adjusted for relative mutation rates (SeattleSNP data)^a^

CpG	Substitution	5' UTR	3' UTR	Rad	Cons	Syn	Introns
nonCpG	C:G > A:T	0.0	25.6	4.1	12.9	15.2	23.7
nonCpG	C:G > G:C	0.0	17.9	4.7	10.6	12.1	30.0
nonCpG	C:G > T:A	13.8	23.4	4.0	5.9	10.3	22.5
CpG	C:G > A:T	0.0	0.0	0.5	1.0	4.3	6.8
CpG	C:G > G:C	0.0	26.0	1.2	0.0	2.8	22.8
CpG	C:G > T:A	7.7	49.5	2.1	2.0	6.0	55.0
nonCpG	A:T > C:G	28.5	20.5	2.7	8.7	4.0	22.9
nonCpG	A:T > G:C	8.3	20.9	3.6	6.8	8.0	23.8
nonCpG	A:T > T:A	0.0	19.9	2.2	1.5	0.0	19.1

^a^All values × 10^5^

## Discussion

The absolute number of potential sites in the coding region was estimated on the basis of the frequency of codons from which a given type of substitution originated and on the size of the coding region. We further subdivided the functional categories into nine mutation types on the basis of ancestral and derived alleles. This allowed us to estimate the effects of mutability (relative mutation rates) and selection (functional categories) on SNP density.

We found that the mutation rate was strongly correlated with PSS. We assumed that different functional categories are under varying selection pressures; for example, radical missense mutations are under stronger purifying selection than are conservative and synonymous mutations. After adjusting PSSs by mutation rates, we found that functional type had a significant effect on SNP density (*P *= 0.02), suggesting that selection also plays a role in SNP density. This result is consistent with those of other studies [11,14,17,19,42].

To estimate the relative effect of mutability and selection on the proportion of segregating sites we compared the model in which both mutability and selection were used as predictors with the models in which either mutability or functional category alone were the predicting variables and found that approximately 87% of PSS variation was due to variation in the mutation rate and 13% was explained by the variation in selection intensity (functional categories). The finding that mutation rate rather than selection plays a major role in SNP density may be important for the design of association studies. Case-control association studies are widely used to identify genetic variants that affect susceptibility to common human diseases [43-46]. An association study usually identifies the candidate gene (or region) and may require resequencing of the candidate region to identify functional SNPs. Resequencing of the whole candidate region might be too expensive; therefore, it is important to identify regions that are most likely to contain functional polymorphisms. Our results show that the adjusted PSSs in the 5' UTRs are similar to, or even lower than, the PSSs for radical missense mutations.

Analysis of the SeattleSNPs data produced results that support the idea that 5' UTR regions in the human genome are subject to strong purifying selection. The adjusted PSS for 5' UTR SNPs was similar to that for radical missense mutations and was significantly lower compared to the intronic and 3' UTR SNPs. In contrast to analysis of the dbSNP database, we found no differences between synonymous, conservative and radical substitutions. This may be a result of a much smaller sample size and therefore insufficient power to detect the differences. It is also possible that the analysis based on the SeattleSNP data is biased. The genes for the SeattleSNPs project were selected based on the two criteria: i) the gene should be functionally important with strong evidence for its involvement in common human diseases; and ii) the gene should be relatively small to allow the direct sequencing. It is possible, therefore, that the Seattle genes are under a stronger pressure of purifying selection compared to an average gene in the human genome. This also could explain our finding that based on the dbSNP data, the ratio of the adjusted PSS for 3' UTR to the PSS for radical missense mutations was 3.4, but it was 18.5 based on the SeattleSNPs data.

### Limitations of the analysis

Our analysis provides a bird eye view of the control of PSS in exonic regions of the human genome. Exonic regions are more likely to be targeted for SNP discovery compared to the intronic and intergenic regions [47,48], the reason why we excluded intronic and intergenic regions from our dbSNP-based analysis. We did not take into account the fact that intensity of purifying selection can be different for different genes or different regions in the gene. The reason why we did not analyze more detailed functional categories is that the density of SNPs in the human genome is currently not sufficient to conduct the gene-centered analysis.

Our analysis provides only rough estimates of the effect of mutability. Though we took into account major sources of variation in the mutation rates – transitional or transversional type of substitution and CpG sites [there was almost 50-fold difference in the relative mutations rate in our study (Table 1)], it still may be not sufficiently detailed because there is growing evidence that local differences in nucleotide composition can lead to up to 4-fold differences in the mutation rate [49-51]. We limited our analysis to the 9 basic mutational categories because further categorization of sites by mutation rate would unlikely provide statistically robust estimates.

Ancestral allele information in our analysis was derived from the comparison of human and chimpanzee sequences. It is based on the assumption that the outgroup sequence is identical to the ancestral sequence. Multiple mutations during species divergence may violate this assumption leading to misidentification of the ancestral allele [52]. About 2.6% SNPs in the coding regions of the human genome may be misidentified [53]. Misidentification is expected to be highest for sites with the highest mutation rate and because the derived alleles tend to be rare, the misidentification will mislabel rare allele as common leading to inflation of non-neutrality tests, especially those designed to detect positive selection. It is hard to estimate the effect of misidentification of ancestral alleles on the proportion of segregating sites.

We estimated the number of the potential sites in CpG dinucleotides by summarizing the CpGs in codons and CpGs located on codon boundaries. To our knowledge, there are only two studies that address the CpG frequencies at codon boundaries. Analysis of hemoglobin genes [54] demonstrated almost two-fold excess of CpGs at codon boundaries. On the contrary, a more recent study of 369 genes (author did not indicate criteria were used for the gene selection) from tomato demonstrated a more than two-fold deficit of CpGs compared to the expected based on the frequency of Cs at third and frequency of Gs on the first codon position [55]. Analysis of CpGs in Seattle genes demonstrated a 3-fold deficit of the CpG dinucleotides at codon boundaries. After the adjustment for the deficit of CpG at codon boundaries, the proportion of CpG dinucleotides was ~0.03 in our analysis, which is close to the proportion of CpGs – 0.028 estimated based on the analysis of genes from the Santa Cruz human genome assembly (hg16) [56].

## Conclusion

In conclusion, our results suggest that 5' UTRs are under purifying selection pressure as strong as that affecting radical missense mutations. This is consistent with the results reported by Osada *et al*. [57], who compared 169 human and macaque gene sequences and detected a much lower rate of substitution in 5' UTRs than the rate of synonymous substitutions. The authors concluded that SNPs in 5' UTR regions are subject to purifying selection. These findings and ours suggest that 5' UTRs are important sites to identify functional disease-associated polymorphisms.

## Supplementary Material

Additional file 1 Estimation of the relative mutation rates. The data provided describe how the relative mutation rates for 9 mutational categories were estimated. Click here for file

Additional file 2 The proportions of the various functional/mutational categories of SNPs in the genome, based on codon usage. The data provided describe how the number of potential sites for functional/mutational categories was estimated. Click here for file
